# Tortoise Plastron and Deer Antler Gelatin Prevents Against Neuronal Mitochondrial Dysfunction *In Vitro*: Implication for a Potential Therapy of Alzheimer’s Disease

**DOI:** 10.3389/fphar.2021.690256

**Published:** 2021-05-13

**Authors:** Dan Cheng, Xin-Jing Yang, Lu Zhang, Zong-Shi Qin, Wen-Qi Li, Hai-Chun Xu, Zhang-Jin Zhang

**Affiliations:** ^1^ School of Chinese Medicine, LKS Faculty of Medicine, The University of Hong Kong, Hong Kong, China; ^2^ Department of Chinese Medicine, The University of Hong Kong-Shenzhen Hospital (HKU-SZH), Shenzhen, China; ^3^ Shenyang Jing’an Mental Health Hospital, Shenyang, China

**Keywords:** deer antler gelatin, tortoise plastron gelatin, Alzheimer’s disease, oxidative damage, mitochondrial dysfunction

## Abstract

Mitochondrial dysfunction with oxidative damage plays the fundamental roles in the pathogenesis of Alzheimer’s disease. In traditional Chinese medicine (TCM) practice, animal tissue-derived gelatins are often used as nootropic agents to treat cognitive deterioration and senile dementia. Tortoise plastron gelatin (TPG) and deer antler gelatin (DAG) are the two most commonly used gelatins for this purpose. This study sought to examine the effects of the two gelatins in preventing neuronal mitochondria from oxidative damage. PC12 cells, a cell line derived from rat pheochromocytoma, exposed to the neurotoxin Aβ_25–35_ served as an *in vitro* model of Alzheimer’s disease. The cells were separately pre-treated with TPG and DAG at various concentrations ranging from 6.26 µg/ml–200 µg/ml, followed by co-incubation with 20 μM Aβ_25–35_ for different duration. Cell viability, mitochondrial membrane potential (MMP) and ultrastructure, intracellular ATP, reactive oxygen species (ROS) and calcium (Ca^2+^) level, the expression of mitochondrial dynamic proteins and biomarkers of apoptosis were measured. Pretreatment with TPG and DAG reversed the Aβ-induced reduction of cell viability in a dose-dependent manner. Both TPG and DAG significantly increased MMP and ATP, alleviated the accumulation of damaged mitochondrial fragments, and normalized the aberrant expression of multiple mitochondrial dynamic proteins of the Aβ-exposed cells. Both gelatins also suppressed intracellular ROS overproduction and Ca^2+^ overload, overexpression of cytochrome c and pro-apoptosis biomarkers induced by the Aβ exposure. These results suggest that TPG and DAG may have the anti-dementia potential by preventing neuronal mitochondria from oxidative damage.

## Introduction

Alzheimer’s disease (AD) is a progressive neurodegeneration disorder that affects millions of people worldwide. The most apparent pathological feature of AD is the accumulation of amyloid β (Aβ) peptide in the neocortex, hippocampus, and other brain regions essential for cognitive function (Eckert et al., 2010). Aβ is a neurotoxin that impairs subcellular components of cerebral neurons, including mitochondria, leading to neuron degeneration and death (Coleman and Yao, 2003). Due to the fact that the development of multiple drugs directly targeting Aβ has failed to achieve the expected success (Huang et al., 2020), neuronal oxidative hypometabolism has been considered as an alternative therapeutic target in a search of novel therapy for AD (Costantini et al., 2008; Struble et al., 2010).

Mitochondria is the primary site of energy metabolism that plays the fundamental roles in the pathogenesis of AD (Mcbride et al., 2006; Lim et al., 2010). During the evolution of AD, mitochondria suffer profound alterations, manifesting as morphological damage, decreased ATP generation, and increased production of reactive oxygen species (ROS) (Lin and Beal, 2006). An excessive ROS production, in turn, exacerbates mitochondrial dysfunction with decreased mitochondrial membrane potential (MMP) and intracellular calcium (Ca^2+^) overflow (Manczak et al., 2010; Tönnies and Trushina, 2017), resulting in the release of apoptotic factors, such as cytochrome c, an apoptotic mediator in the organelles and procaspase-9 and caspase-3, the two pro-apoptotic factors in response to apoptotic signals (Hengartner, 2000; Giorgi et al., 2012). Neuronal calcium influx further disturbs the maintenance of mitochondrial dynamics with an imbalance in mitochondrial fission and fusion, leading to aberrant expression of fission-mediated proteins, such as fission1 (fis1) and dynamin-related protein 1 (Drp1), and fusion-associated proteins, such as Mitofusin 1 (Mfn1), Mitofusin 2 (Mfn2), and Optic atrophy 1 (Opa1) (Ishihara et al., 2004; Losón et al., 2013).

In traditional Chinese medicine (TCM) practice, animal tissue-derived gelatins are often empirically used as nootropic and brain function-enhancing agents to treat cognitive deterioration and senile dementia (Kong, 2007; Chen et al., 2016; Zhan et al., 2018; Li and Ms, 2019; Tang et al., 2019). Tortoise plastron gelatin (TPG) and deer antler gelatin (DAG) are the two most commonly prescribed gelatins for this purpose (Li and Ms, 2019; Tang et al., 2019). Previous studies have revealed that TPG and DAG contain multiple amino acids and trace minerals, and possess the effects in modulating energy metabolism and improving memory and other cognitive symptoms (Table 1; Shi et al., 2012; Jiang et al., 2015; Guo et al., 2016; Wang et al., 2018; Long et al., 2019). These have led to the hypothesis that the anti-dementia effects of the two gelatins were perhaps achieved by preventing neuronal mitochondria from oxidative damage. To test this hypothesis, the effects of TPG and DAG were examined in Aβ_25–35_-exposed PC12 cells, a cell line derived from a rat pheochromocytoma, that served as an *in vitro* model of Alzheimer’s disease. It is well documented that PC12 is an appropriate model that has been widely used to investigate mitochondria-and Alzheimer’s disease-related pathological profiles (Dubey et al., 2019). Cell viability, mitochondrial ultrastructure and MMP, intracellular ATP, ROS and Ca^2+^ level, the expression of mitochondrial dynamic-and apoptosis-related biomarkers were measured.

**TABLE 1:** Amino acids and trace mineral elements contained in the gelatins^a^.

Components	Tortoise plastron gelatin	Deer antler gelatin
Amino acids (%)		
Alanine	4.5–8.2	6.8–9.8
Arginine	2.4–5.7	2.4–15.0
L-Aspartic acid	2.1–2.3	2.2–7.2
L-Cystine	0.2–0.3	0.2–1.0
Glutamic acid	3.5–7.6	3.1–12.4
Glycine	14.3–18.2	17.1–55.9
Histidine	0.2–0.4	0.4–1.3
Hydroxyproline	7.6–7.9	8.8–11.9
Isoleucine	0.7–1.8	1.3–3.0
Leucine	1.5–2.8	2.7–5.3
Lysine	1.4	1.5–5.8
Phenprobamate	1.4–1.9	2.0–3.9
Proline	6.7–11.8	8.5–19.2
Serine	2.4–3.0	2.5–2.6
Threonine	1.0–1.6	1.1–2.5
Tyrosine	0.5–0.8	0.6
Valine	0.9–1.6	1.6–4.5
Trace mineral elements (mg/kg)		
Calcium (Ca)	150–961	
Magnesium (Mg)	115–760	
Zinc (Zn)	0.3–06	
Iron (Fe)	36–122	
Strontium (Sr)	0.1–34.3	

a Data are generated from Refs [Guo et al. (2016), Long et al. (2019), Wang et al. (2018), Jiang et al. (2015), Shi et al. (2012)].

## Materials and Methods

### Drug Preparations

Tortoise plastron gelatin (TPG) and deer antler gelatin (DAG) were generously provided by Hongxing pharmaceutical Co. Ltd (Shantou, Guangdong, China). TPG-and DAG-contained products have been approved by National Medical Products Administration for marketing (Approved code: Z44021133) (http://gdhxjtgfyxgshxzyc.liangyi360.com/yaopin/818003604.html). The detailed manufacturing procedure of the two gelatins was carried out in strict compliance with Pharmacopoeia of the People’s Republic of China (2020) and Good Manufacturing Practice (GMP). The quality control of both gelatins adhered to the specifications and test procedures according to the internal standard. The gelatins were dissolved into phosphate buffered saline (PBS) to form a 20 mg/ml stock solution and stored at 4°C for later use.

Aβ_25–35_ fragments (Sigma-Aldrich) were generated as described in a previous method (Kim et al., 2010). Briefly, Aβ_25–35_ monomer was dissolved in distilled water to a stock concentration of 1 mmol/L, incubated at 37°C for seven days, and then stored Aβ_25–35_ plaques at-20°C for later use.

### Cell Culture and Treatment

PC12 cells were cultured in Dulbecco’s Modified Eagle Media (DMEM, Gibco, America) supplemented with 10% fetal bovine serum (FBS, Gibco, United States) and 1% Penicillin-Streptomycin Solution (Invitrogen, Unites States) at 37°C in a 5% CO_2_ incubator.

The cells were seeded into 96-well plates at a density of 5 × 10^3^ cells/well for cell viability analysis, 10 cm cell culture dish for transmission electron microscopy and 6-well plates at a density of 1.25 × 10^5^ cell/ml for other experiments. Seeded cells were allowed to grow for 24 h. After then, for most experiments, the cells were treated respectively with multiple concentrations of TPG and DAG, ranging from 6.26 µg/ml–200 µg/ml, for 1 h; 20 μM Aβ_25–35_ was subsequently added for co-incubation for additional 23 h, such that the preventive effects of the gelatins against Aβ neurotoxicity was tested. Following the completion of treatment, various experimental procedures were further performed.

A separate experiment was conducted to examine the acute effects on MMP (see below) by adding 20 μM Aβ_25–35_ at 20 h after gelatin treatment and co-incubating for additional 4 h. To determine the optimal time point at which the production of Aβ-induced intracellular (ROS) reached the peak level, the cells were exposed to 20 μM Aβ_25–35_ for 1, 6, 12, and 24 h, respectively. The highest ROS level was observed at 1 h (Supplementary Figure S2). The ROS experiment was then conducted by adding 20 μM Aβ_25–35_ at 23 h after gelatin treatment and co-incubating for additional 1 h (see below).

### Cell Viability Assay

Cell viability was measured using a Cell Counting Kit-8 (CCK-8) assay (ApeBio, United States). Briefly, following the completion of treatment, 10 μl CCK-8 solution was added to each well and incubated for 2 h. The absorbance for optical density (OD) in each well was read at 450 nm on a Microplate reader (Bio-Rad, United States). Cell viability was calculated using the following equation: Cell viability (%) = (mean OD of drug-treated cells-mean OD blank)/(mean OD of untreated cells-mean OD blank) × 100%.

### Transmission Electron Microscopy

The cells were collected into the trypsin-EDTA solution, centrifuged at 1,000 rpm for 5 min, washed for three times in PBS, and fixed in a 2.5% glutaraldehyde solution at 4°C overnight. After then, glutaraldehyde solution was removed. The cells were washed for three times in PBS, post-fixed in 1% osmium tetroxide for 3 h and stained with 2% uranyl acetate at 4°C. The cells were further dehydrated in a gradient ethanol concentration from 50 to 100% and embedded in spur resin. Ultrathin (60 nm) sections were stained in a combination of 3% uranyl acetate and lead citrate, and then examined under TEM (Philips CM100).

### Flow Cytometry Analysis

Flow cytometry was used to analyze MMP and cell apoptosis.

MMP was examined using a JC-1 mitochondrial assay kit (Beyotime, Beijing, China) with flow cytometry (BD, United States). JC-1 forms aggregates that emit red fluorescence at high MMP while forms monomers that emit green fluorescence at low MMP (Berlepsch and Böttcher, 2013). PC12 cells were washed twice in PBS and incubated at 37°C for 30 min at the final concentration of JC-1 (1×) dissolved in the staining buffer and distilled water. The cells were washed three times in PBS and re-suspended in the same buffer in the culture tube for flow cytometry detection.

Cell apoptosis was analyzed using PE Annexin V Apoptosis detection kit (BD Pharmingen, United States), in which Annexin V was conjugated to the fluorochrome phycoerythrin (PE), to detect the phosphatidylserine (PS) on the outer membrane of the apoptosis cells (Leventis and Grinstein, 2010). PC12 cells were washed twice with cold PBS and then re-suspended in 1× binding buffer at a concentration of 1 × 10^6^ cell/mL. After that, 100 μL of cells were transferred to the culture tube and incubated with a mixture of 10 μl PE Annexin V and 7-AAD at room temperature for 20 min in the dark. Cells were subjected to flow cytometric analysis.

### Fluorescence Spectrophotometry of ATP

The intracellular ATP level was measured using a luminescent ATP detection assay kit (Beyotime, Beijing China). Following the treatment as described above, the medium was discarded and the cells were washed for three times in PBS. Then, 200 μl lysis buffer of luminescent ATP assay was added to each well and incubated for 30 min. The cells were collected and centrifuged at 12,000 g for 5 min; the supernatant was aspirated for cell lysate. Finally, 90 μl ATP assay kit was added to 10 μl of cell lysate or the ATP standard solution in the 96-well plates. Luminescence was measured with fluorospectrometer.

### Fluorescence Analysis of Intracellular Ca^2+^and ROS Levels

The intracellular Ca^2+^ level was measured using the Fluo-4-AM (Invitrogen, United States) dye. PC12 cells were stained with 2.5 μM of Fluo-4-AM solution for 30 min in darkness at 37°C and then washed for three times in Hank’s Balanced Salt Solution (HBSS) (Corning, United States). Green fluorescence, which reflects the intracellular Ca^2+^ level, was recorded with a fluorescence microscope (Carl Zeiss, Germany).

Intracellular ROS levels were detected using fluorescence microscopy with DCFH-DA (Beyotime Biotechnology, Beijing, China). The DCFH-DA was intracellularly deacetylated by a nonspecific esterase, further oxidized by ROS to produce the fluorescent compound 2,7-dichlorofluorescein (DCF) (An et al., 2017). The analysis was performed with the assay kit protocol. Briefly, PC12 cells were washed twice in DMEM without FBS and incubated with DCFH-DA at 37°C for 25 min. The cells were then washed twice with PBS and analyzed in a fluorescence microscope (Carl Zeiss, Germany).

The intensity of Ca^2+^ and ROS-labelled cells was further quantified. The labeled cells were counted from five areas under × 20 field or × 40 field randomly chosen from each well using ImageJ software.

### Western Blot Analysis

PC12 cells were lyzed in radioimmunoprecipitation assay (RIPA) buffer (Sigma-Aldrich, United States) supplemented with a protease inhibitor cocktail (Cell Signaling Technology, United States). The total protein quantification and western blot procedures were performed routinely (Bass et al., 2017). Equal amounts of proteins were resolved by sodium dodecyl sulfate-polyacrylamide gel electrophoresis (SDS-PAGE) and transferred onto polyvinylidene difluoride (PVDF) membranes. The membranes were incubated with blocking buffer (5% BSA) for 1.5 h at first and then incubated with the primary antibodies against mitochondrial dynamic-related proteins, Drp-1, Mfn1, Mfn2, Fis1, p-Drp1 (ser616), and Opa1 (Proteintech, Wuhan, China); the antibodies against apoptosis-related biomarkers, caspase-9, cleaved caspase-3, cytochrome c (CST, United States), caspase-3 (Santa-Cruz Biotechnology, Unites States), Bax (Abclonal), and Bcl-2 (Abcam, USA); the Ca^2+/^calmodulin-dependent protein kinases, CaMKII, p-CaMKII (Santa-Cruz Biotechnology, USA). After that, the corresponding secondary antibodies were added on PVDF membrane for additional incubation for 1.5 h at room temperature. Finally, the proteins were detected using an ECL select western blotting detection reagent (GE Healthcare, United States) and quantified in ChemiDoc XRS + imaging system (Bio-Rad, Unites States).

### Statistical Analysis

All data were expressed as mean ± standard error (SE). One-way analysis of variance (ANOVA) was used to detect statistical significance, followed by Tukey’s HSD post hoc test to determine between-group differences. *p* < 0.05 was defined as statistical significance. Statistical analyses were performed using the GraphPad prism 6 (Graphpad software Inc., Califonia, United States).

## Results

### Cell Viability

**FIGURE 1:**
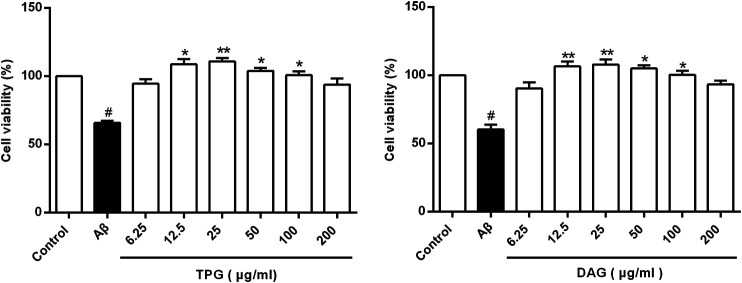
Cells viability of the Aβ_25–35_-exposed cells being treated with TPG and DAG. Statistical analyses were performed using one-way ANOVA and Tukey-HSD post hoc comparisons. All values were shown as mean ± SEM from three independent studies. #*p* < 0.05, vs. control group; *p < 0.05, vs. Aβ_25–35_ models; **p < 0.01, vs. models. TPG, tortoise plastron gelatin; DAG, deer antler gelatin.

The viability of the Aβ_25–35_-exposed cells was pronouncedly lower than that of the unexposed cells (*p* = 0.0078). TPG-and DAG-pretreated cells at concentration of 12.5–100 µg/ml exhibited significantly greater viability than the Aβ_25–35_-exposed cells (*p* < 0.05). The peak effects of the two gelatins was present consistently at 25 µg/ml. This concentration was then selected for further experiments (Figure 1). IC_50_, the concentration that produces a 50% reduction of cell viability, was 2,173 µg/ml with TPG and 2,430 µg/ml with DAG, which were 87- to 97-fold higher than that of the peak effects.

### The Effects on Mitochondrial Ultrastructure

**FIGURE 2:**
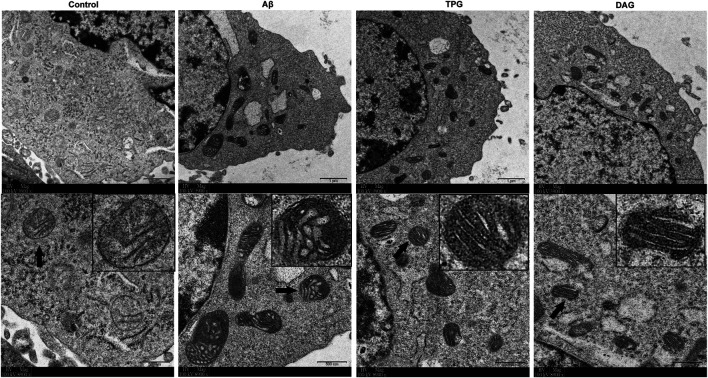
The improvement of TPG and DAG on mitochondrial ultrastructure in the Aβ_25–35_-exposed cells. The transmission electron microscope (TEM) was used to examine ultrathin slices or in different groups. The images below each group were the enlarged representative mitochondria. The representative mitochondria marked by black arrow in each group were magnified in the black box. Magnification: ×3,900 and ×8,900, respectively. Scale bars: 1 μm and 500 nm, respectively. TPG, tortoise plastron gelatin; DAG, deer antler gelatin.

The Aβ exposure caused a large accumulation of damaged mitochondrial fragments with excessive fission and fusion, unclearly arranged crests, and defective organelle morphology. The cells pre-treated with TPG and DAG displayed the neatly arranged lamellar mitochondrial crests (Figure 2).

### The Effects on Intracellular ATP Level

**FIGURE 3:**
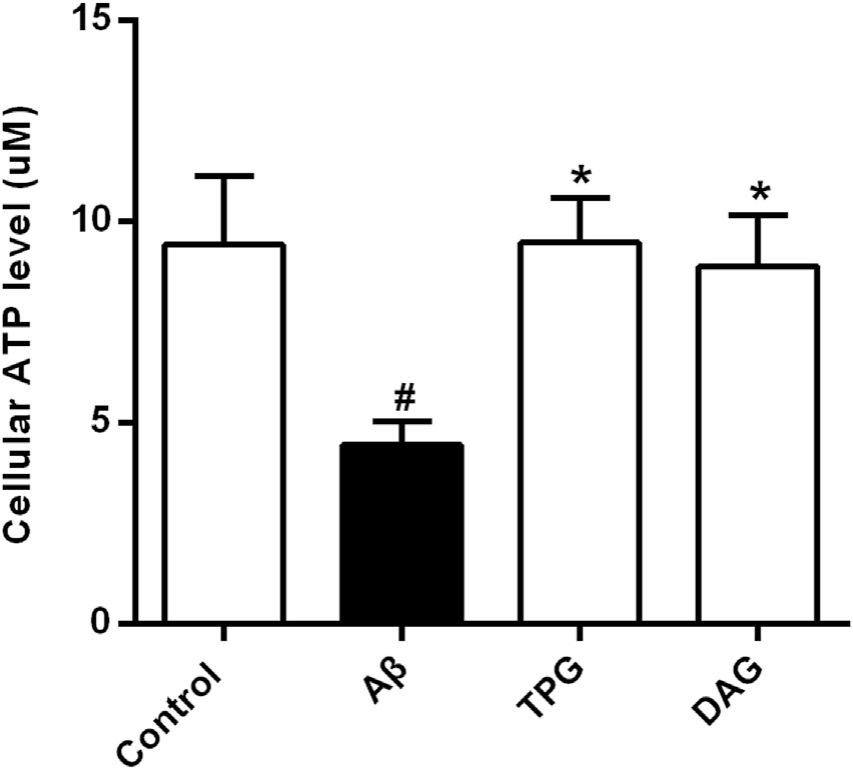
The improvement of TPG and DAG on the intracellular ATP level in Aβ_25–35_-exposed cells. The concentrations of intracellular ATP level in different groups. All values were shown as mean ± SEM from three independent studies. Statistical analyses were performed using one-way ANOVA and Tukey-HSD post hoc comparisons. ^#^ *p* < 0.05, vs. control group; ^*^ *p*＜0.05, vs. Aβ_25–35_ models. TPG, tortoise plastron gelatin; DAG, deer antler gelatin.

Intracellular ATP level of the Aβ_25–35_-exposed cells was 4.5 ± 0.6 μM. This was markedly lower than 9.4 ± 1.7 μM of the untreated cells (*p* = 0.0214), 9.5 ± 1.1 μM of the TPG-treated cells (*p* = 0.0203), and 8.9 ± 1.3 μM of the DAG-treated cells (*p* = 0.0345). The levels of the latter three groups were not significantly different (Figure 3).

### The Effects on Mitochondrial Membrane Potential

**FIGURE 4:**
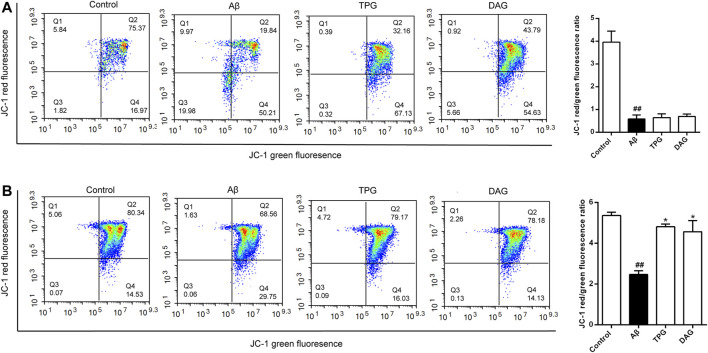
The improvement of TPG and DAG on mitochondrial membrane potential in Aβ_25–35_-exposed cells. Cells with high MMP promote the formation of dye aggregates and fluoresce red, while cells with low potential contain monomeric JC-1 and fluoresce green. The ratio of red/green fluorescence indicated MMP level in each group **(A)** The ratio of red/green fluorescence in different groups after 24 h of Aβ_25–35_-exposed cells **(B)** The ratio of red/green fluorescence in different groups after 4 h of Aβ_25–35_-exposed cells. All values were shown as mean ± SEM from three independent studies. Statistical analyses were performed using one-way ANOVA and Tukey-HSD post hoc comparisons. ^#^ *p* < 0.05, vs. control group; ^##^ *p* < 0.01, vs. control group; ^*^ *p* ＜ 0.05, vs*.* Aβ_25–35_ models. TPG, tortoise plastron gelatin; DAG, deer antler gelatin.

The Aβ_25–35_ exposure for 4 and 23 h caused an approximately two-and six-fold decrease of MPP, respectively, as compared with the unexposed cells (*p* < 0.01) (Figure 4). MPP value of both TPG-and DAG-pretreated cells was strikingly greater than that of the cells co-incubated with Aβ_25–35_ for 4 h (*p* < 0.05) (Figure 4A), but similar to that co-incubated for 23 h (Figure 4B).

### The Effects on Reactive Oxygen Species

**FIGURE 5:**
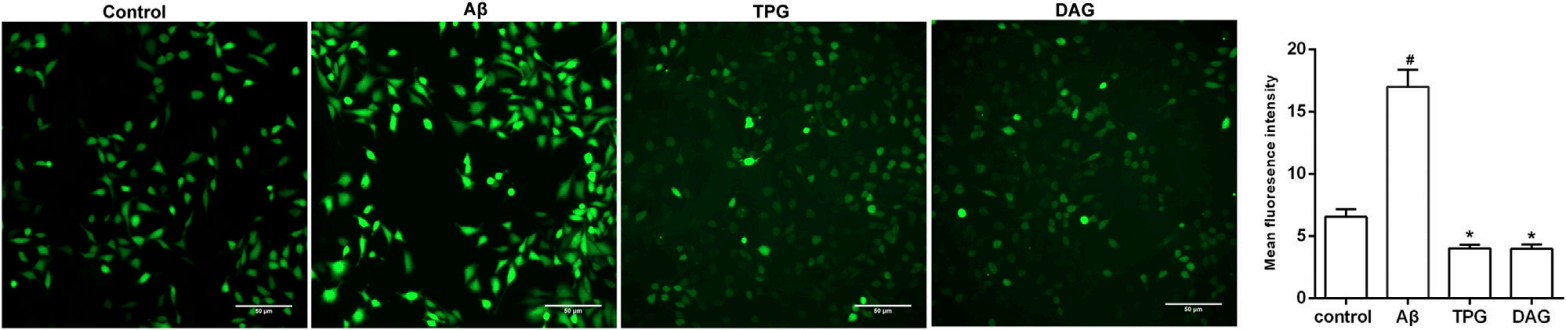
The regulation of TPG and DAG on the intracellular ROS in Aβ_25–35_-exposed cells. The intracellular ROS level was recorded by fluorescence microscope with DCFH-DA. The intracellular ROS level in different groups after being induced with Aβ_25–35_ or treated with the two gelatins. Scale bars: 50 μm. All values were shown as mean ± SEM. #*p* < 0.05, vs. control group; *p＜0.05, vs. Aβ_25–35_ models. TPG, tortoise plastron gelatin; DAG, deer antler gelatin.

The intracellular ROS level was measured at 1, 6, 12, and 24 h after Aβ_25–35_ exposure. The production of intracellular ROS reached the peak level at 1 h post-exposure, but gradually decreased with increasing exposure time (Supplementary Figure S2). The effects of TPG and DAG on ROS were thus examined in the 1 h exposed cells. The intracellular ROS level of TPG-and DAG-treated cells was strikingly lower than that of the unexposed and Aβ_25–35_-exposed cells (*p* < 0.005) (Figure 5).

### The Effects of on Intracellular Ca^2+^ and CaMKII

**FIGURE 6:**
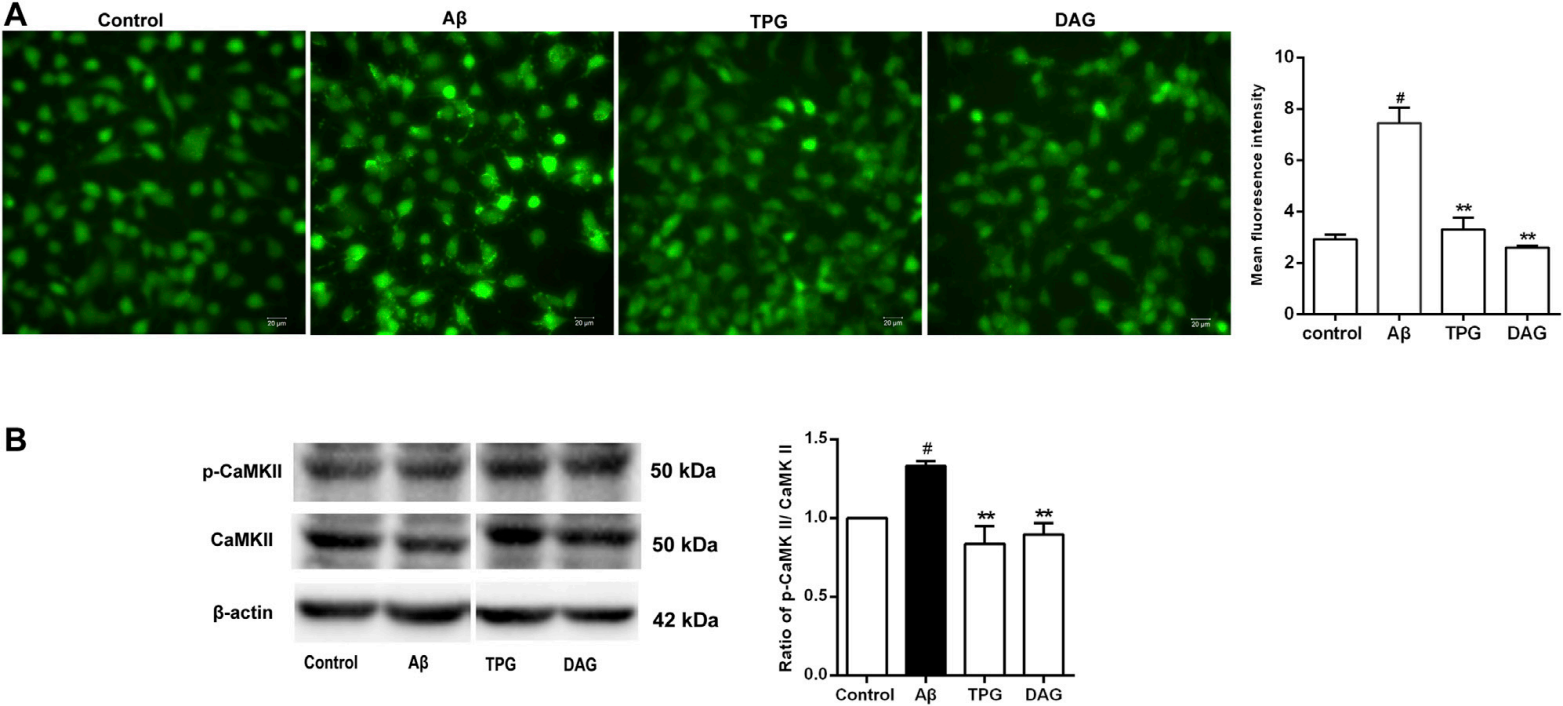
The regulation of TPG and DAG on the calcium overload in the Aβ_25–35_-exposed cells **(A)** The image of intracellular calcium in different groups recorded by a fluorescence microscope. Scale bars: 20 μm **(B)** The expression level of CaMKII and its phosphorylation in different groups were measured by western blot. All values were shown as mean ± SEM from three independent studies. Statistical analyses were performed using one-way ANOVA and Tukey-HSD post hoc comparisons. ^#^ *p* < 0.05, vs. control group; *p＜0.05, vs. Aβ_25–35_ models; **p＜0.01, vs. Aβ_25–35_ models. TPG, tortoise plastron gelatin; DAG, deer antler gelatin.

Intracellular Ca^2+^ level of the Aβ_25–35_-exposed cells was significantly higher than that of the unexposed cells (*p* = 0.0051). Pre-treatment with TPG and DAG remarkably lowered the Aβ-induced increase of Ca^2+^ (*p* < 0.01) (Figure 6A).

The expression of CaMKII, a downstream calcium protein, and its phosphorylation (p-CaMKII), was examined using western blot. The ratio of p-CaMKII/CaMKII was calculated. The Aβ_25–35_ exposure caused a significant increase of the ratio compared to the unexposed cells (*p* = 0.0378). The cells pre-treated with TPG and DAG markedly reduced the increased ratio to control value (*p* < 0.01) (Figure 6B).

### The Effects on the Expression of Mitochondrial Dynamic-Related Proteins

**FIGURE 7:**
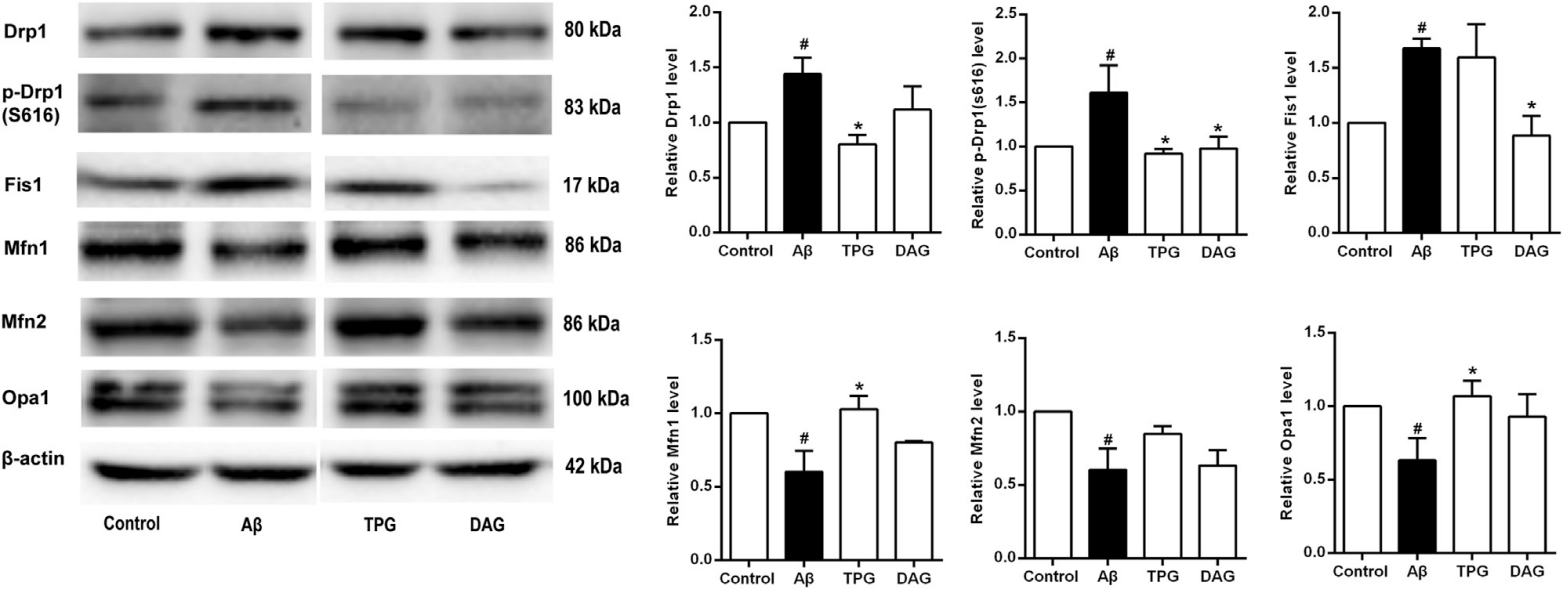
The regulation of TPG and DAG on the expression level of mitochondrial dynamics-related proteins in Aβ_25–35_-exposed cells. The expression level of the proteins in each group was relative to the control group. All values were shown as mean ± SEM from three independent studies. Statistical analyses were performed using one-way ANOVA and Tukey-HSD post hoc comparisons. ^#^ *p* < 0.05, vs. control group; ^*^ *p*＜0.05, vs. Aβ_25–35_ models. TPG, tortoise plastron gelatin; DAG, deer antler gelatin.

The expression of Drp1 and Fis1, the two proteins that regulate the mitochondrial fission, and the phosphorylation of Drp1 at ser616 [p-Drp1 (ser616)] was detected. The Aβ_25–35_ exposure induced significant increase of the expression of the three fission proteins compared to the unexposed cells (*p* < 0.05). Pre-treatment with TPG reversed the Aβ_25–35_-induced increase of the Drp1 and p-Drp1 (ser616) expression (*p* < 0.05), but had no effects on Fis1. Pre-treatment with DAG markedly suppressed the overexpression of Drp1 (ser616) and Fis1 (*p* < 0.05), but did not change the overexpression of Drp1 (Figure 7).

The three mitochondrial fusion-related proteins, Mfn1, Mfn2, and Opal, was also evaluated. The expression level of the three proteins of the Aβ_25–35_-exposed cells was markedly lower than that of the unexposed cells (*p* < 0.05). The TPG-pretreated cells had significantly higher level of Mfn1 and Opa1 than the Aβ_25–35_-exposed cells (*p* < 0.05). DAG had no effects on the Aβ_25–35_-induced decrease of the three fusion protein expression (Figure 7).

### The Effects on Cell Apoptosis and Related Biomarkers

**FIGURE 8:**
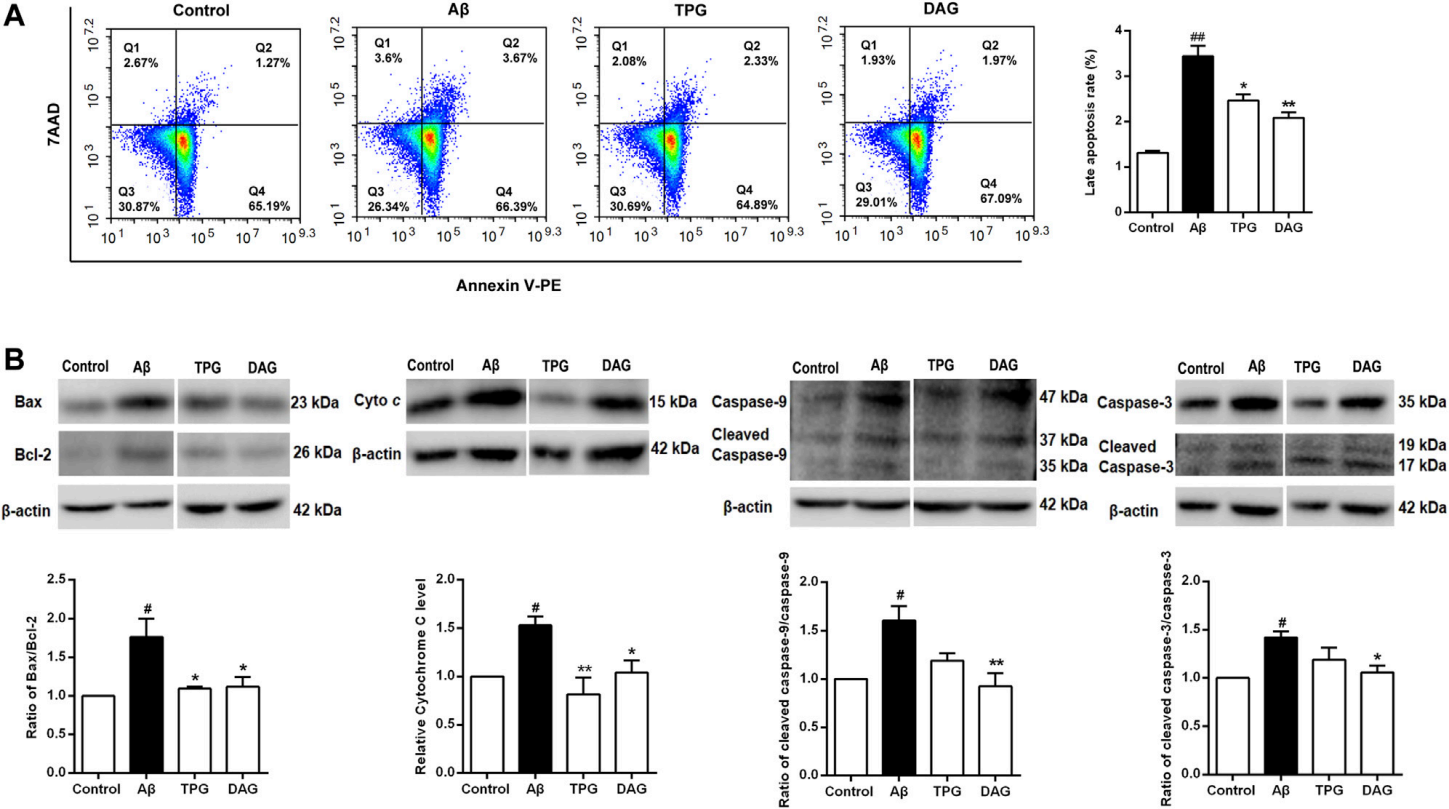
The regulation of TPG and DAG on the cell apoptosis **(A)** The apoptotic cell was measured by flow cytometry. Late apoptotic cell rate was detected in different groups after 20 h pre-treatment with the four drugs and a subsequent 4 h incubated with Aβ_25–35_ **(B)** The expression levels of pro-apoptosis proteins were detected using western blot. All values were shown as mean ± SEM from three independent studies. Statistical analyses were performed using one-way ANOVA and Tukey-HSD post hoc comparisons. *p* ^#^ < 0.05, vs. control group; ^*^ *p* ＜ 0.05, vs. Aβ_25–35_ models; ^**^ *p*＜0.01, vs. Aβ_25–35_ models. Cyto c, Cytochrome c; TPG, tortoise plastron gelatin; DAG, deer antler gelatin.

Cell apoptosis was examined using flow cytometry. Percent of late apoptotic cells was strikingly increased in the Aβ_25–35_-exposed cells (*p* = 0.0018). Pretreatment with TPG and DAG prominently reduced percent of the late apoptosis cells (*p* < 0.05) (Figure 8A).

The expression of the pro-apoptotic proteins, cleaved-caspase-9, caspase-9, cleaved-caspase-3, caspase-3, and cytochrome c, and the apoptosis regulators, Bax and Bcl-2, was evaluated with western blot. The ratios of cleaved-caspase-9/caspase-9, cleaved-caspase-3/caspase-3, and Bax/Bcl-2 were obtained. The Aβ_25–35_-exposed cells exhibited the overexpression of cytochrome c (*p* = 0.0377) and significant increases of the three ratios (*p*< 0.05). Pretreatment with both gelatins significantly decreased the ratio of Bax/Bcl-2 and the expression of cytochrome c (P < 0.05). DAG further significantly reduced the ratios of cleaved-caspase-9/caspase-9 and cleaved-caspase-3/caspase-3 (*p* < 0.05) (Figure 8B).

## Discussion

This study represents the first to explore novel anti-dementia agents from animal tissue-derived gelatins that may have the potential in preventing against neuronal mitochondrial dysfunction. Previous studies have confirmed that Aβ toxicity-induced multiple molecular and subcellular pathological events were initially triggered by the impairment of mitochondrial Ca^2+^ buffering capacity, leading to an intracellular overflow of Ca^2+^, damaged mitochondrial respiratory chain, and excessive production of ROS (Orrenius et al., 2003; Manczak et al., 2011; Pallo and Johnson, 2015). This study revealed that the Aβ_25–35_ exposure resulted in a dramatic increase of intracellular Ca^2+^ level, rapid overproduction of ROS and cellular and mitochondrial damage, manifesting as reduced cell viability, mitochondrial fragmentation, increased cytochrome c level, and decreased MMP. The neurotoxin also largely inhibited mitochondrial ATP production. These results well validated the Aβ_25–35_-exposed PC12 cells as an *in vitro* model in mimicking Alzheimer’s disease in association with mitochondria dysfunction.

We further found that the intracellular ROS level reached the peak in an hour, but gradually decreased to a minimum level in 24 h following the Aβ_25–35_ exposure. Likewise, MMP decreased from 2-fold at 4 h to 6-fold at 24 h. Similar phenomena were also observed in previous studies, confirming that oxidative stress occurred in the early stage of Alzheimer’s disease could mediate oxygen-containing substances and generate ROS (Sancar et al., 2004; Fan et al., 2017). It thus appears that the Aβ-induced excessive production of ROS is an early, short-term, but irreversible event that subsequently causes the disruption of MMP, leading to mitochondrial DNA damage and functional defects (Wang et al., 2008).

Pretreatment with TPG and DAG suppressed the Aβ_25–35_-induced increase of Ca^2+^ and cytochrome c, and prevented cell viability and mitochondrial ultrastructure from Aβ toxicity. The two gelatins completely resumed decreased ATP production to the control level. Both TPG and DAG also reversed MMP to the control values in the short-term Aβ_25–35_ exposure, although they had no effects in preventing MMP from the long-term Aβ_25–35_ exposure. Both gelatins further inhibited the rapid overproduction of ROS. These results suggest that TPG and DAG could protect mitochondrial structure and functions by blocking intracellular Ca^2+^ overflow and early excessive production of ROS. Additionally, this study also indicated the least cytotoxic effects of the two gelatins, as evidenced by the fact that IC_50_ values were average 92-fold higher than the concentration of the peak effects.

One apparent molecular pathology of mitochondria caused by Aβ is the disturbance of mitochondrial dynamics, manifesting as an imbalance in the expression of mitochondrial fission and fusion proteins that are involved in the mitochondrial division and maintenance of mitochondrial morphology (Taguchi et al., 2007; Manczak et al., 2011; Itoh et al., 2013; Kim et al., 2016; Pernas and Scorrano, 2016; Jiang et al., 2018). In particular, the phosphorylation of Drp1-serine 616 is essential in the mitochondrial division (Taguchi et al., 2007). In this study, we observed that the expression of Drp1, p-Drp1 (s616), and Fis1 was increased, but the expression of Mfn1, Mfn2, and Opal was decreased in the Aβ_25–35_-exposed cells. These results proved the effects of the neurotoxin in causing aberrant expression of mitochondrial fission and fusion proteins. While both TPG and DAG equivalently inhibited the phosphorylation of Drp1-serine 616 and had no effects on Mfn2, TPG displayed stronger effects in normalizing the aberrant expression of Drp1, Mfn1, and Opa1, whereas DAG only had a significant effect in suppressing Fis1. It seems to suggest that TPG and DAG may differentially mediate mitochondrial dynamic proteins, i.e., in addition to fission proteins, TPG also had significant effects in mediating fusion proteins, but DAG did not. Whether a combination of the two gelatins with the differential effects on the dynamic proteins could produce additive or even synergistic actions in preventing mitochondrial functions deserves for further investigations.

Mitochondria dynamic proteins interact with apoptosis and Ca^2+^ homeostasis. The downregulation of Drp1 and Fis1 attenuated the release of cytochrome c and delayed apoptosis (Iwasawa et al., 2011; Clerc et al., 2014). Mfn2 plays a crucial role in the conduction of calcium signals (Wang et al., 2015). On the other hand, the Aβ-induced intracellular Ca^2+^ overload activated the downstream proteins, calmodulin-dependent protein kinase II (CaMKII), then upregulated the phosphorylation of Drp1-serine 616, and increased the fission event rates (Xu et al., 2016; Divakaruni et al., 2018). In this study, while the two gelatins exerted differential effects on the dynamic proteins, they inhibited intracellular Ca^2+^ overflow and the phosphorylation of CaMKII induced by Aβ. It appears that the effects of the gelatins in protecting mitochondria function may be associated with their mediation of the interaction between mitochondrial dynamic proteins and calcium-dependent pathways.

Aβ-induced mitochondrial Ca^2+^ overload further triggered mitochondrial Ca^2+^-dependent apoptosis by opening the mitochondrial permeability transition pore (mPTP) and releasing cytochrome c and pro-apoptotic factors, such as caspase-9 and caspase-3 (Joza et al., 2001; Orrenius et al., 2003; Abramov et al., 2007; Chen et al., 2011; Calvo Rodriguez et al., 2020). Bax is a pro-apoptotic protein that causes the loss of MMP and eventually lead to the release of apoptotic factors into the cytoplasm, while Bcl-2 inhibits cell apoptosis (Glantz et al., 2006; Ola et al., 2011). The relative level of Bax and Bcl-2 is thus essential for determining whether cells undergo cell apoptosis (Khodapasand et al., 2015). This study revealed that the Aβ_25–35_-exposed cells had a much higher proportion of late apoptotic cells, increased ratio of Bax/Bcl-2, cleaved-caspase-9/caspase-9, and cleaved-caspase-3/caspase-3. Aβ_25–35_ also induced the overexpression of cytochrome c. These results confirm the effects of Aβ_25–35_ in inducing apoptosis *via* the mitochondrial pathway. Pretreatment with TPG and DAG, however, decreased the proportion of late apoptotic cells, the ratio of Bax/Bcl-2 and the expression level of cytochrome c. DAG further suppressed the Aβ_25–35_-induced increase of ratios of cleaved-caspase-9/caspase-9 and cleaved-caspase-3/caspase-3. The preventive effects of the two gelatins appear to be, at least partly, derived from their inhibition of mitochondrial apoptosis pathway.

Given that Ca^2+^ dyshomeostasis is heavily involved in the pathogenesis of Alzheimer’s disease, agents that regulate Ca^2+^ homeostasis and protect mitochondrial function may possess anti-dementia potential (Calvo Rodriguez and Bacskai, 2020). It has been reported that endogenous and exogenous cations and amino acids indirectly regulates the calcium-sensing receptor (CaSR), a classic G protein-coupled receptor, and maintain Ca^2+^ homeostasis in the body. In this process, amino acids act as pure positive allosteric modulators to enhance calcium-active receptor function (Liu et al., 2020). As shown in Table 1, TPG and DAG contain an abundance of various amino acids and trace mineral elements (Shi et al., 2012; Jiang et al., 2015; Guo et al., 2016; Wang et al., 2018; Long et al., 2019). It seems that the two gelatins act as positive allosteric regulators to enhance the efficiency of the calcium-active receptor, thereby activating CaSR, maintaining intracellular calcium homeostasis, and protecting mitochondrial function. On the other hand, glycine, the most abundant amino acid of the two gelatins, could protect against brain injury by regulating mitochondria-mediated autophagy (Cai et al., 2019). Lower level of iron, manganese, and zinc have been observed in patients with Alzheimer’s disease (Basun et al., 1991). Iron, zinc, calcium, manganese, and strontium level correlated with memory and cognitive functions in patients with Alzheimer’s disease (Basun et al., 1991; Veronese et al., 2016; Lei et al., 2021). It is suggested that TPG and DAG could serve as specific supplements for the treatment of Alzheimer’s disease.

There are three apparent limitations in this study. First, this was a preliminary *in vitro* study. Behavioral, pharmacological, and *in vivo* imaging approaches in animal models should be further considered to determine the anti-dementia or nootropic effects. Second, although the batch of TPD and DAG used in this study was not examined, it was manufactured in compliance with Pharmacopoeia of the People’s Republic of China (2020) and Good Manufacturing Practice (GMP). The contents of major components have been well identified with high consistency as shown in Table 1 (Shi et al., 2012; Jiang et al., 2015; Guo et al., 2016; Wang et al., 2018; Long et al., 2019). Despite this, there is no global standard available for the preparation of the two gelatins. Caution should nevertheless be taken when other TPD and DAG products are used. Third, various amino acids and trace mineral elements are contained in the two gelatins. As these small-molecule compounds are more accessible to the blood–brain barrier (BBB), they could exert greater pharmacokinetic and pharmacodynamic effects in the central nervous system compared with the macromolecular natural compounds, such as ginsenosides. Further experiments should be considered to identify major components that may play the principal roles in the therapeutic effects of the two gelatins.

## Conclusion

Pretreatment with TPG and DAG reversed the Aβ_25–35_-induced decrease in cell viability, MMP and ATP, alleviated the accumulation of damaged mitochondrial fragments, and normalized the aberrant expression of multiple mitochondrial dynamic proteins of the Aβ_25–35_-exposed cells. Both gelatins also suppressed intracellular ROS overproduction and Ca^2+^ overload, overexpression of cytochrome c and pro-apoptosis biomarkers induced by the Aβ_25–35_ exposure. This study suggests that TPG and DAG may possess the anti-dementia potential and could serve as specific supplements for the treatment of Alzheimer’s disease.

## Data Availability

The original contributions presented in the study are included in the article/Supplementary Material, further inquiries can be directed to the corresponding author.

## Abbreviations

TPG, Tortoise plastron gelatin; DAG, deer antler gelatin; AD, Alzheimer’s disease; MMP; mPTP, mitochondrial membrane permeability transition pores; Drp1, dynamin-related protein 1; Fis1, fission 1; Mfn1, mitofusin l; Opa 1, optic atrophy 1; TCM, traditional Chinese medicine; GMP, Good Manufacturing Practice; ChP, Pharmacopoeia of the People’s Republic of China; PBS, phosphate-buffered saline; DMEM, Dulbecco’s Modified Eagle Media; FBS, Fetal bovine serum; CCK-8, Cell Counting Kit; TEM, Transmission electron microscope; HBSS, Hank’s Balanced Salt Solution; DCF, 2,7-dichlorofluorescein; RIPA, radioimmunoprecipitation assay; SDS-PAGE, sodium dodecyl sulfate-polyacrylamide gel electrophoresis; PVDF, polyvinylidene difluoride; TBST, Tris-buffered saline containing Tween 20; CaMKII, calmodulin-dependent protein kinase II; CaSR, calcium-sensing receptor.
