# Supplementary figures and images for: Tortoise Plastron and Deer Antler Gelatin Prevents Against Neuronal Mitochondrial Dysfunction In Vitro: Implication for a Potential Therapy of Alzheimer’s Disease
Source: Front Pharmacol. 2021 May 13;12:690256. doi: 10.3389/fphar.2021.690256 (PMC8155591; doi:10.3389/fphar.2021.690256)

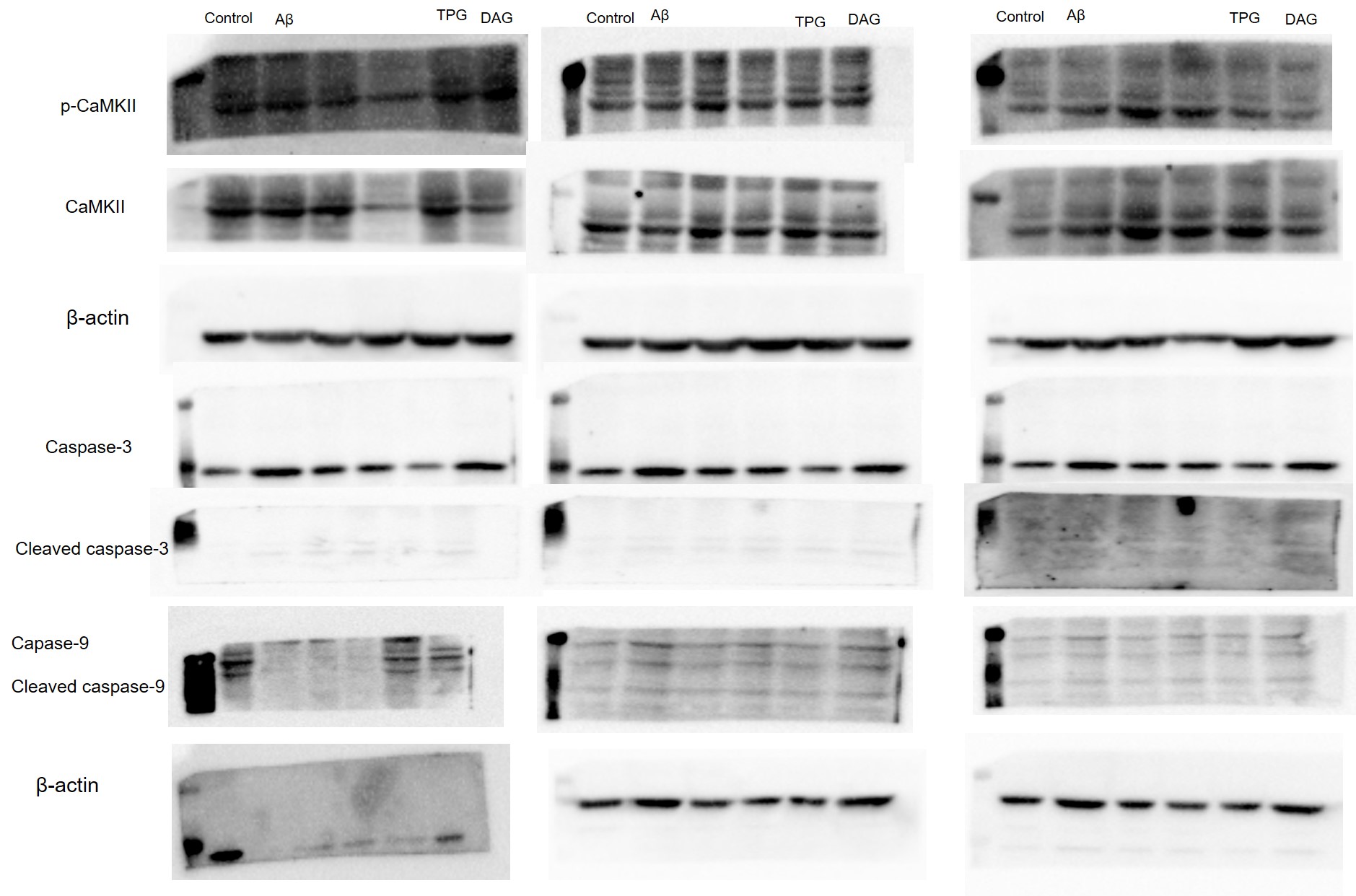

Supplement: Supplementary file 1 [file Image3.JPEG]

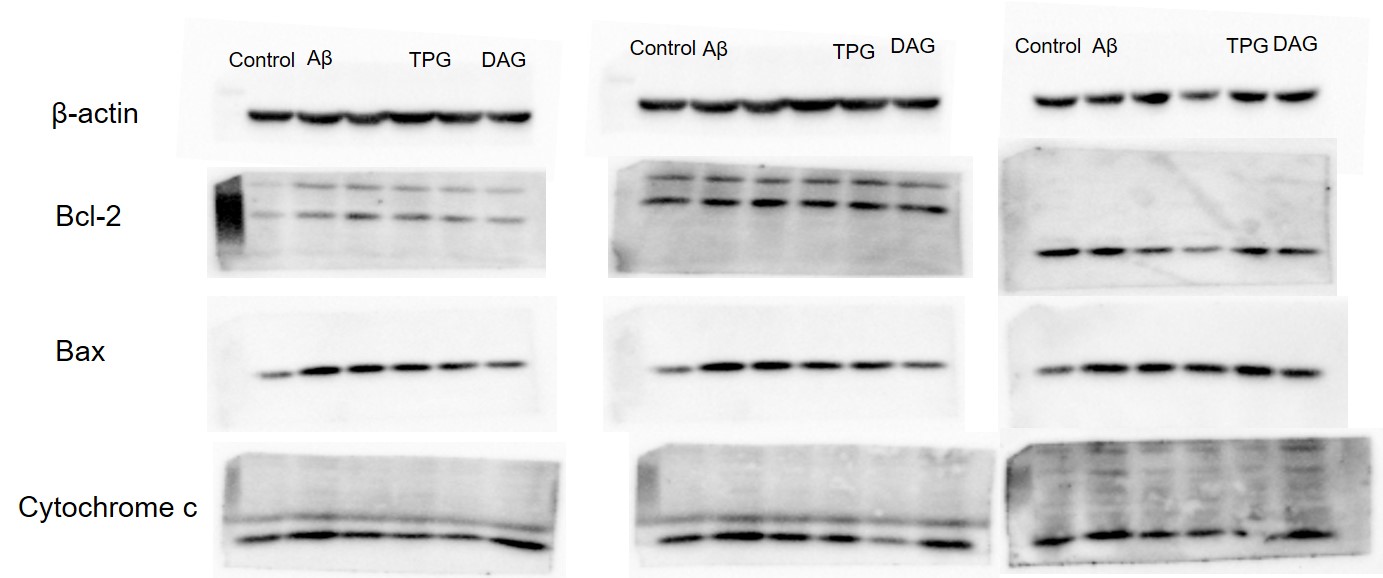

Supplement: Supplementary file 2 [file Image1.JPEG]

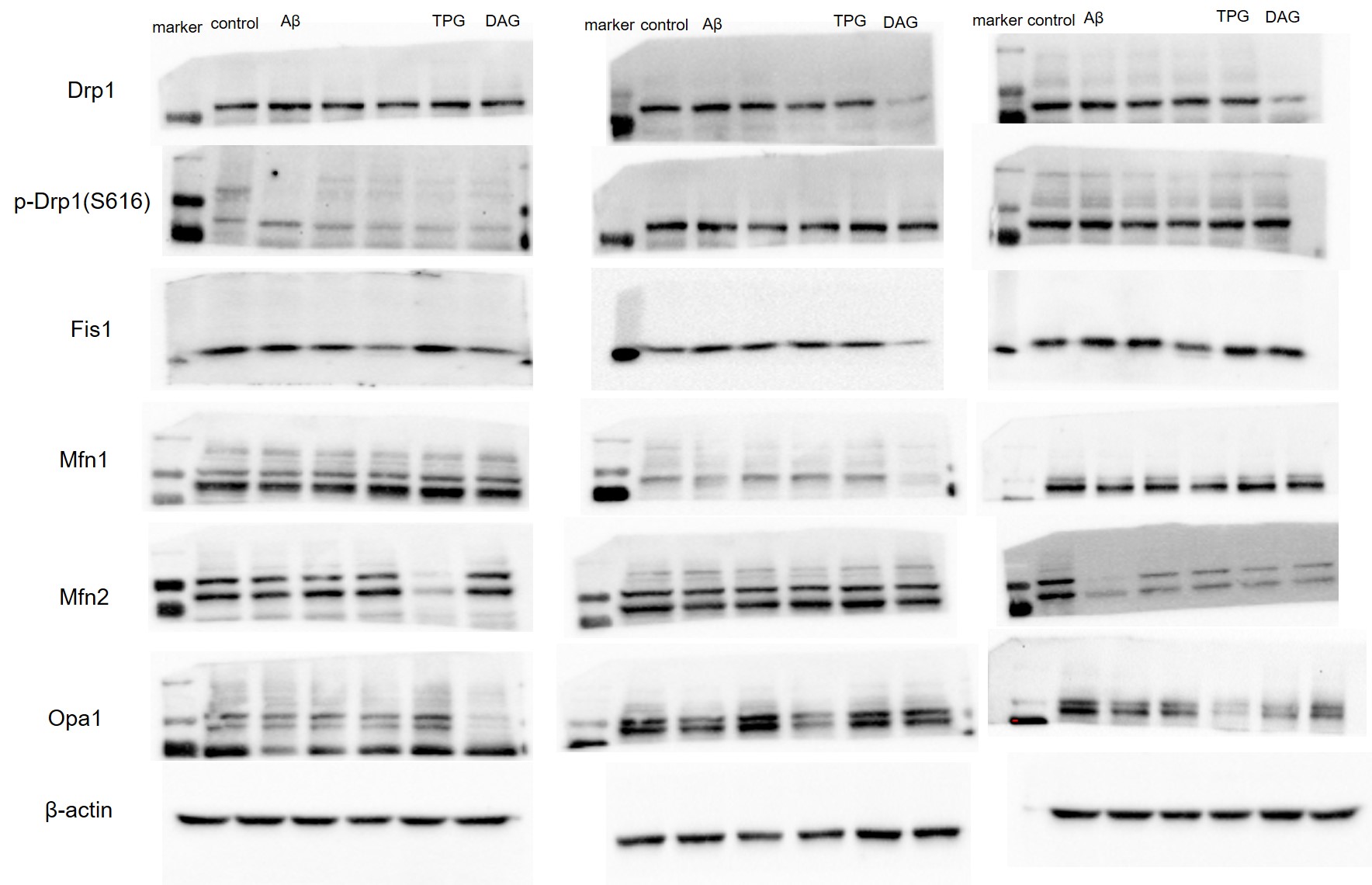

Supplement: Supplementary file 3 [file Image2.JPEG]
