# Supplementary material for: Tortoise Plastron and Deer Antler Gelatin Prevents Against Neuronal Mitochondrial Dysfunction In Vitro: Implication for a Potential Therapy of Alzheimer’s Disease
Source: Front Pharmacol. 2021 May 13;12:690256. doi: 10.3389/fphar.2021.690256 (PMC8155591; doi:10.3389/fphar.2021.690256)
Supplement: Supplementary file 4 [file DataSheet1.pdf]

# Supplementary material

## Cell Viability Assay

PC12 cells were seeded into the 96-well plates at a density of $5 \times 10^3$ cells /well. After 24 hours, cells were pre-treated with series concentrations of the two gelatins from 1-1000 µg/mL for 1 hour, and then 20 µM of $A\beta_{25\text{-}35}$ was added and incubated with the drugs for another 23 hours. Cell viability in response to the different treatments was measured using a CCK-8 assay kit. Briefly, after treatment, 10 µL of Cell Counting Kit (CCK-8) solution (ApeBio, USA) was added to each well and incubated for 2 hours. The absorbance in each well was read at 450 nm on a Microplate reader (Bio-Rad, USA). Cell viability was calculated using the following equation: Cell viability (%) = (Mean OD drug-treated cells- Mean OD blank) / (Mean OD untreated cells-Mean OD blank) × 100 %. The results showed that the cell viability increased significantly when the concentration was 10 and 100 µg/ml.

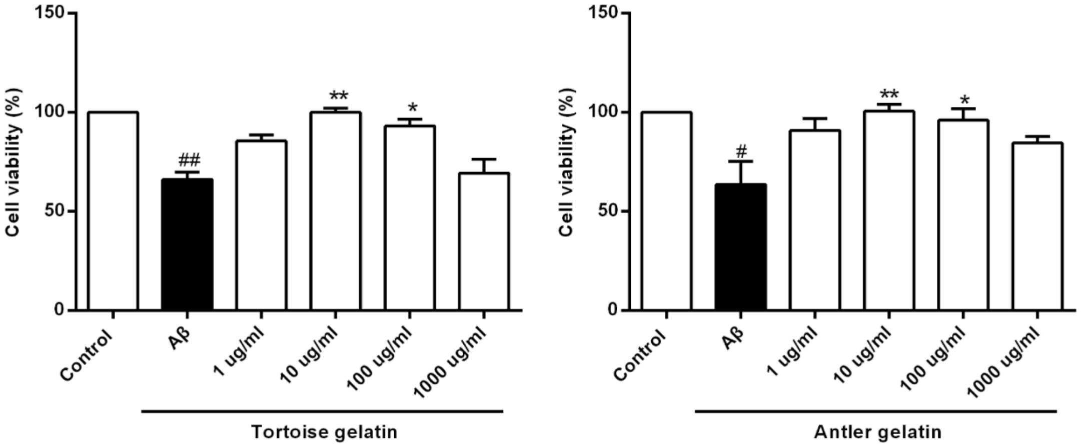


Figure 1S. Cells viability of the Aβ-exposed cell being treated with tortoise plastron gelatine and dear antler gelatine. Statistical analyses were performed using one-way ANOVA and Tukey-HSD post hoc comparisons. $^{\#}p < 0.05$, *vs.* control group; $^{\#\#}p < 0.01$, *vs.* control group;* $p < 0.05$, *vs.* $A\beta_{25\text{-}35}$ models; ** $p < 0.01$, *vs.* $A\beta_{25\text{-}35}$ models. Tortoise gelatin: tortoise plastron gelatin; Antler gelatin: dear antler gelatin.

## Intracellular ROS level

To determine the optimal time point at which the production of $A\beta_{25\text{-}35}$-induced intracellular reactive oxygen species (ROS) reached the peak level, the cells were

exposed to 20 μM A$\beta_{25-35}$ for 1, 6, 12, and 24 hours, respectively. The analysis was performed with the DCFH-DA (Beyotime Biotechnology, Beijing, China) assay kit protocol. Briefly, PC12 cells were washed twice in DMEM without FBS and incubated with DCFH-DA at 37°C for 25 min. The cells were then washed twice with PBS and analyzed in a fluorescence microscope (Carl Zeiss, Germany). The production of intracellular ROS reached the peak level at 1 hour after the A$\beta_{25-35}$ exposure, and the mean fluorescence intensity of A$\beta_{25-35}$-exposed cells was about three times that of normal cells at this time point (P=0.0267). While the mean fluorescence intensity gradually decreased with increasing exposure time. The intracellular ROS level at 24 hours was almost the same as even lower than that in the normal cells. (Supplementary Fig. 2S).

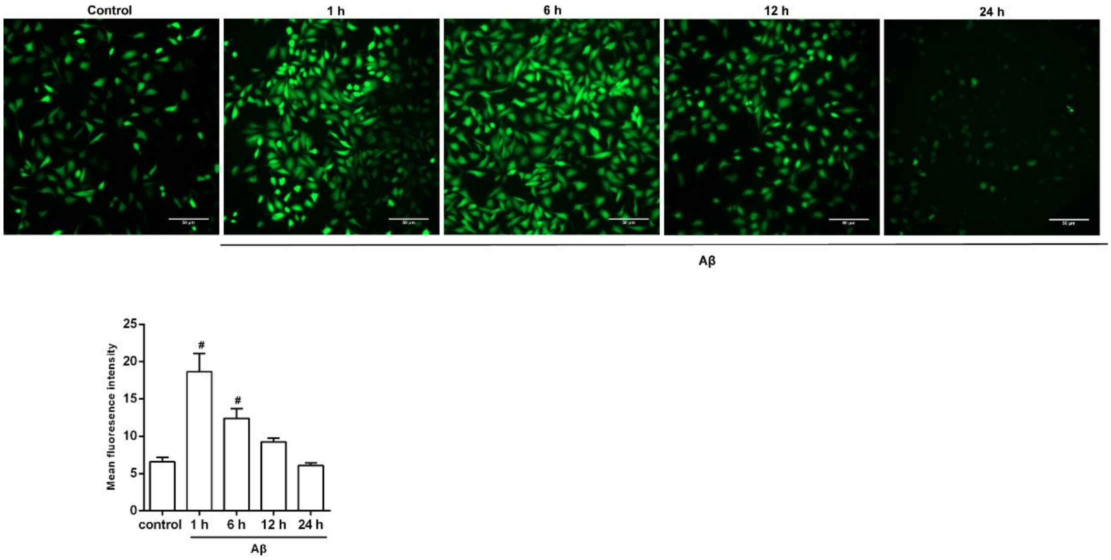


Figure 2S. The fluorescence intensity of A$\beta_{25-35}$-exposed cells at different time points. Scale bar: 50 μm. All values were shown as mean ± SEM. Statistical analyses were performed using one-way ANOVA and Tukey-HSD post hoc comparisons. $^{\#}p < 0.05$, *vs*. control group.
